# Removal of Phenol by Ozonation in Strongly Alkaline Conditions using a Jet Loop Reactor Operated in Continuous Phase

**DOI:** 10.1002/gch2.202500085

**Published:** 2025-07-02

**Authors:** Melahat Semin Barlak, Ibrahim Cengiz, Nejdet Degermenci, Ergun Yildiz

**Affiliations:** ^1^ Department of Civil and Environmental Engineering Colorado State University Fort Collins CO 80523 USA; ^2^ Faculty of Applied Science Department of Emergency Aid and Disaster Management Atatürk University Erzurum 25400 Türkiye; ^3^ Faculty of Engineering and Architecture Department of Environmental Engineering Kastamonu University Kastamonu 37150 Türkiye; ^4^ Faculty of Engineering Department of Environmental Engineering Atatürk University Erzurum 25240 Türkiye

**Keywords:** chemical oxygen demand, jet loop reactor, ozonation, phenol, total organic carbon

## Abstract

In this study, phenol removal by ozonation under strong alkaline conditions in a continuously operated jet loop reactor (JLR) is investigated. The effects of inlet ozone gas concentration, hydraulic retention time (HRT), and influent phenol concentration on phenol, chemical oxygen demand (COD), and total organic carbon (TOC) removal in the JLR effluent are evaluated. When the inlet ozone gas concentration is 17.5 gO_3_ m^−3^, the steady‐state phenol, COD, and TOC removal efficiencies are determined as 97.8%, 61.1%, and 32.2%, respectively. When the inlet ozone concentration increases from 17.5 to 56.5 gO_3_ m^−3^, phenol is not detected in the JLR effluent. The system operates at different HRTs, and the highest removal efficiency at steady‐state is obtained at 8 h HRT. While phenol is completely removed at this HRT, COD and TOC removals are 76.8% and 48.2%, respectively. An increase in phenol concentration in the JLR influent leads to a reduction in the phenol, COD, and TOC removal efficiencies in the steady‐state effluent.

## Graphical abstract

This study presents the removal of phenol by ozonation in a continuously operated jet loop reactor (JLR) under strong alkaline conditions. The effects of ozone concentration, hydraulic retention time, and influent phenol levels on phenol, COD, and TOC removal are evaluated to assess the efficiency of JLR‐based ozonation.

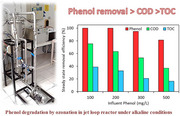

## 1 Introduction

While global demand for water continues to increase due to population growth, industrial development, and improved economic conditions, the number and capacity of accessible water resources continue to decline.^[^ ^1^ ^]^ At the same time, industrial and domestic activities generate large volumes of wastewater, which is often discharged into receiving waters without adequate treatment, causing water quality to deteriorate day by day.^[^ ^2^, ^3^ ^]^ For example, phenols and their derivatives are chemicals found in significant quantities in industrial wastewater and are widely used in everyday life.^[^ ^4^ ^]^ Phenols are discharged to receiving waters in effluents from many industries, including petroleum refining, coking processing, insecticides, paper, pharmaceuticals, plastics, wood, and paint.^[^ ^5^, ^6^ ^]^ Phenols are substances that pose a major risk to humans, animals, and ecological cycles. It is classified as a hazardous pollutant because of its potential to harm human health. Exposure to phenol can increase heart disease, affect the respiratory system, and ingestion can be fatal.^[^ ^7^ ^]^ For this reason, strict discharge standards have been set by international regulatory bodies for the discharge of phenol‐containing wastewater into the receiving environment. According to the Environmental Protection Agency (EPA), the permissible concentration of phenol in surface water should be less than 1 ppb.^[^ ^8^ ^]^ Therefore, industrial wastewater containing phenol should be treated by an effective treatment technology before discharge into the receiving environment. Various treatment methods such as adsorption, photocatalytic degradation, biological treatment, electrochemical oxidation, Fenton oxidation, ozonation have been used for this purpose.^[^ ^9^, ^10^, ^11^, ^12^, ^13^, ^14^ ^]^

Advanced oxidation processes (AOPs) using hydroxyl radicals have been widely used for the treatment of wastewater containing organic matter.^[^ ^15^, ^16^, ^17^, ^18^ ^]^ One of these processes, ozonation, has the advantages of simplicity, strong oxidation, and environmental friendliness.^[^ ^19^ ^]^ In addition, ozonation is used as a pre‐treatment step prior to conventional biological techniques due to its ability to selectively destroy recalcitrant organics, making it more effective for highly contaminated wastewaters.^[^ ^20^ ^]^ However, the use of ozone in wastewater treatment is limited by its poor solubility in water and high production costs. In order to improve the use of ozone and the treatment efficiency of wastewater, an important step is to increase the mass transfer of ozone to the liquid phase.^[^ ^19^ ^]^ For this reason, many reactors have been developed to provide more efficient transfer of ozone gas to water.^[^ ^19^, ^21^, ^22^, ^23^, ^24^ ^]^ The jet loop reactor, as one of the advanced reactor types, demonstrated satisfactory performance with a mass transfer coefficient ranging from 6.1 to 37.3 h^−1^. Although reactors with higher mass transfer coefficients are available, their applicability in large‐scale systems is limited due to their relatively small reactor volumes and high energy requirements.^[^ ^24^ ^]^

Jet loop reactors (JLRs) are known as gas‐liquid contactors.^[^ ^25^ ^]^ These reactors are used for various chemical and biochemical processes as well as wastewater treatment.^[^ ^26^ ^]^ JLRs are known for their high mass transfer capabilities and have simple construction, low investment costs, and relatively low power requirements compared to conventional reactors.^[^ ^27^, ^28^ ^]^ It also has advantages such as more circulation with low energy, high interfacial area between reaction phases, very good gas dispersion, homogeneous concentration, and heat profile. The absence of moving parts in the reactor reduces energy requirements and the need for maintenance and repair. It also has many advantages such as easy transition from pilot to industrial scale.^[^ ^29^, ^30^, ^31^, ^32^, ^33^, ^34^ ^]^ Due to these superior properties, JLRs are attracting increasing interest in chemical processing.^[^ ^31^ ^]^

The reaction of ozone with organic matter in water can occur either directly through reactions of molecular ozone and pollutants, depending on the ozonation conditions, or indirectly through radical reactions (OH radicals) that may result from the decomposition of ozone.^[^ ^35^, ^36^ ^]^ Many studies have indicated that increasing pH improves the formation of hydroxyl radical, which destroys organic compounds more effectively than ozone through the reaction of ozone with hydroxyl ions.^[^ ^37^, ^38^, ^39^ ^]^ Therefore, higher pH means more hydroxyl ions in solution, thus promoting ozone decomposition in solution (Equations 1 and 2) to produce more hydroxyl radicals.^[^ ^39^, ^40^ ^]^ In addition, studies have shown that the reaction rate between phenol and hydroxyl radical (*k* = 2.1 × 10^9^ – 4.5 × 10^9^ M^−1^ s^−1^) is much higher than the reaction rate between phenol and ozone (*k* = 1.3 × 10^3^ M^−1^ s^−1^), and it has been stated that higher phenol degradation rate is obtained with increasing pH.^[^ ^37^ ^]^

(1) $$O_{3}+OH^{-}\to {\mathrm{HO}}_{2}^{-}+O_{2}$$

(2) $$O_{3}+{\mathrm{HO}}_{2}^{-}\to HO^{•}+O_{2}+O_{2}^{-}$$

In this study, a pilot‐scale jet loop reactor (JLR) operated in continuous phase with high mass transfer capacity was used for phenol removal by ozonation under strong alkaline conditions. The effects of ozone gas concentration, hydraulic retention time (HRT), and initial phenol concentration on the changes in phenol, chemical oxygen demand (COD), and total organic carbon (TOC) values in the effluent of the JLR fed with phenol in the continuous phase were investigated.

## 2 Results and Discussion

Phenol‐containing synthetic wastewater prepared from deionized water was fed to the JLR operated in continuous phase. The changes in the phenol, COD, and TOC concentrations at the JLR effluent were monitored. The experiments were started with phenol‐free deionized water in the continuously fed JLR. All experiments were continued until synthetic wastewater containing phenol was passed through the JLR at an average of 6 times the reactor volume. The effect of ozone gas concentration, HRT, and initial phenol concentration on phenol, COD, and TOC removal was investigated.

### 2.1 Effect of Ozone Gas Concentration

**Figure 1:**
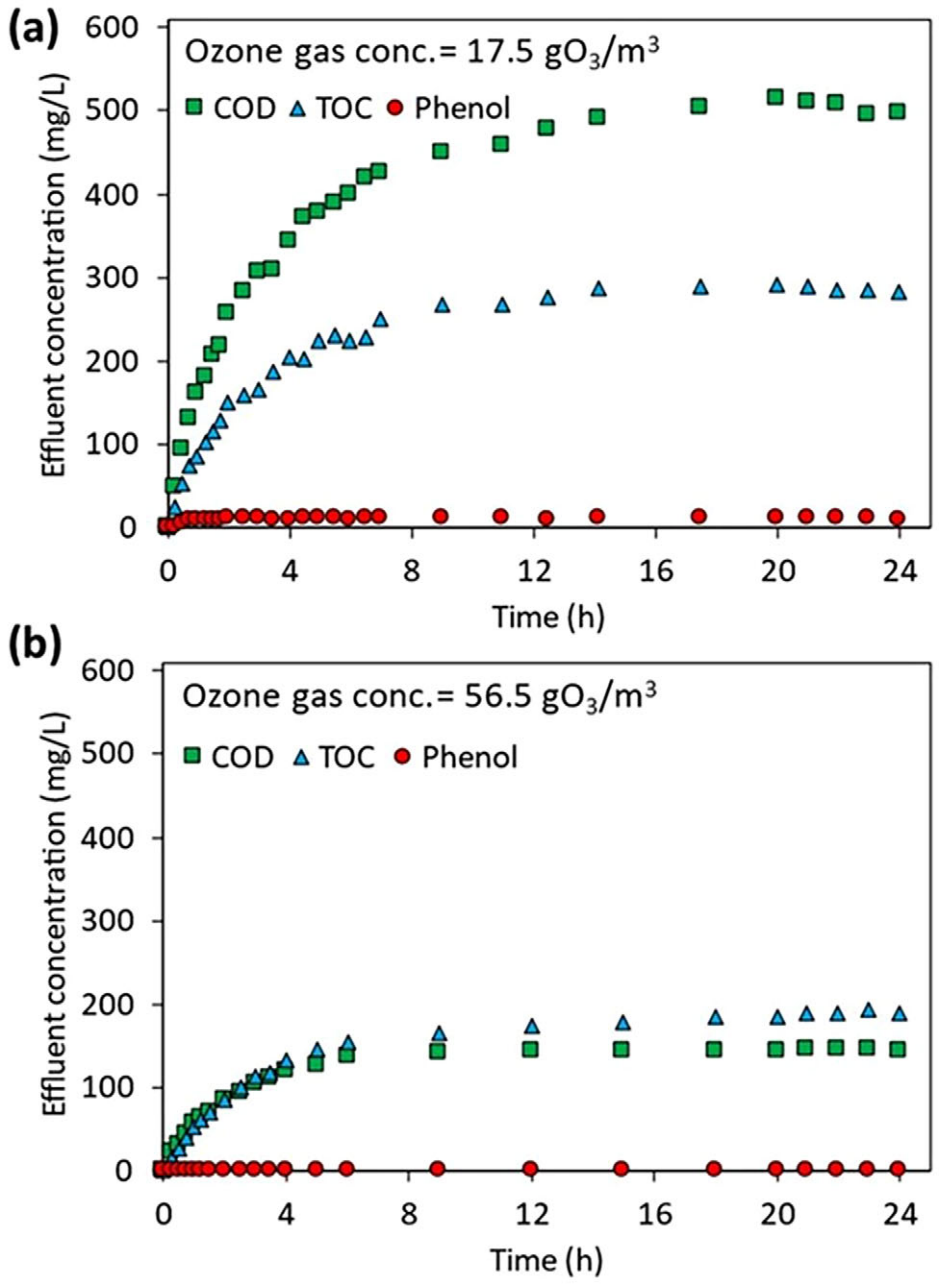
Variation of phenol, COD, and TOC in JLR effluent with time for ozone gas concentrations of a) 17.5 gO_3_ m^−3^ and b) 56.5 gO_3_ m^−3^ (HRT = 4 h, influent phenol = 500 mg L^−1^).

To determine the effect of ozone gas concentration on phenol removal in JLR, two different ozone gas concentrations were studied. In the study, HRT was 4 h and influent phenol concentration was 500 mg L^−1^. The changes in phenol, COD, and TOC values at the JLR effluent with time at different ozone gas concentrations are shown in **Figure** **1**. For an inlet ozone gas concentration of 17.5 gO_3_ m^−3^, the time required for phenol to reach steady‐state at the JLR effluent is ≈2 h, while COD and TOC values require ≈12 h to reach steady‐state (Figure 1a). The steady‐state phenol, COD, and TOC removals in JLR effluent are 97.8%, 61.1% and 32.2%, respectively. As a result of increasing the inlet ozone gas concentration from 17.5 to 56.5 gO_3_ m^−3^, phenol was not detected in the effluent (Figure 1b). At ozone concentration of 56.5 gO_3_/m^3^, approximately 9 hours were required for the COD and TOC values to reach a steady‐state. COD and TOC removal increased with the increase of inlet ozone gas concentration. When the inlet ozone gas concentration was 56.5 gO_3_ m^−3^, COD removal efficiency increased to 88.2% and TOC removal efficiency increased to 53.2% in steady‐state. With the increase in ozone gas concentration, the highest change was obtained in COD value with 27.1%. As the ozone dosage per unit mass of pollutant increased, a notable enhancement in removal efficiency was observed, while the time to reach steady‐state conditions decreased accordingly. Although phenol is completely consumed by ozonation at high pH, it takes longer to remove both COD and TOC due to the formation of intermediate products (muconic, maleic, fumaric, and oxalic acids).^[^ ^41^ ^]^ Oxalic acid, one of the intermediate products formed during the oxidation of phenol, tends to accumulate in ozonated water due to the stability of its chemical structure and its low reaction rate with ozone. The accumulation of oxalic acid makes complete mineralization difficult in ozonation processes.^[^ ^41^, ^42^ ^]^ As a result, oxalic acid remains an important intermediate compound and contributes significantly to the residual organic load measured as COD and TOC. Therefore, although phenol is not observed at the JLR effluent, COD, and TOC originating from intermediate products (mainly oxalic acid) formed due to oxidation are observed. Phenol, COD, and TOC removal efficiencies at the JLR effluent increased with the increase in inlet ozone gas concentration. It was found that the removal efficiencies were in the order of phenol > COD > TOC, regardless of the inlet ozone gas concentration.

### 2.2 Effect of HRT

**Figure 2:**
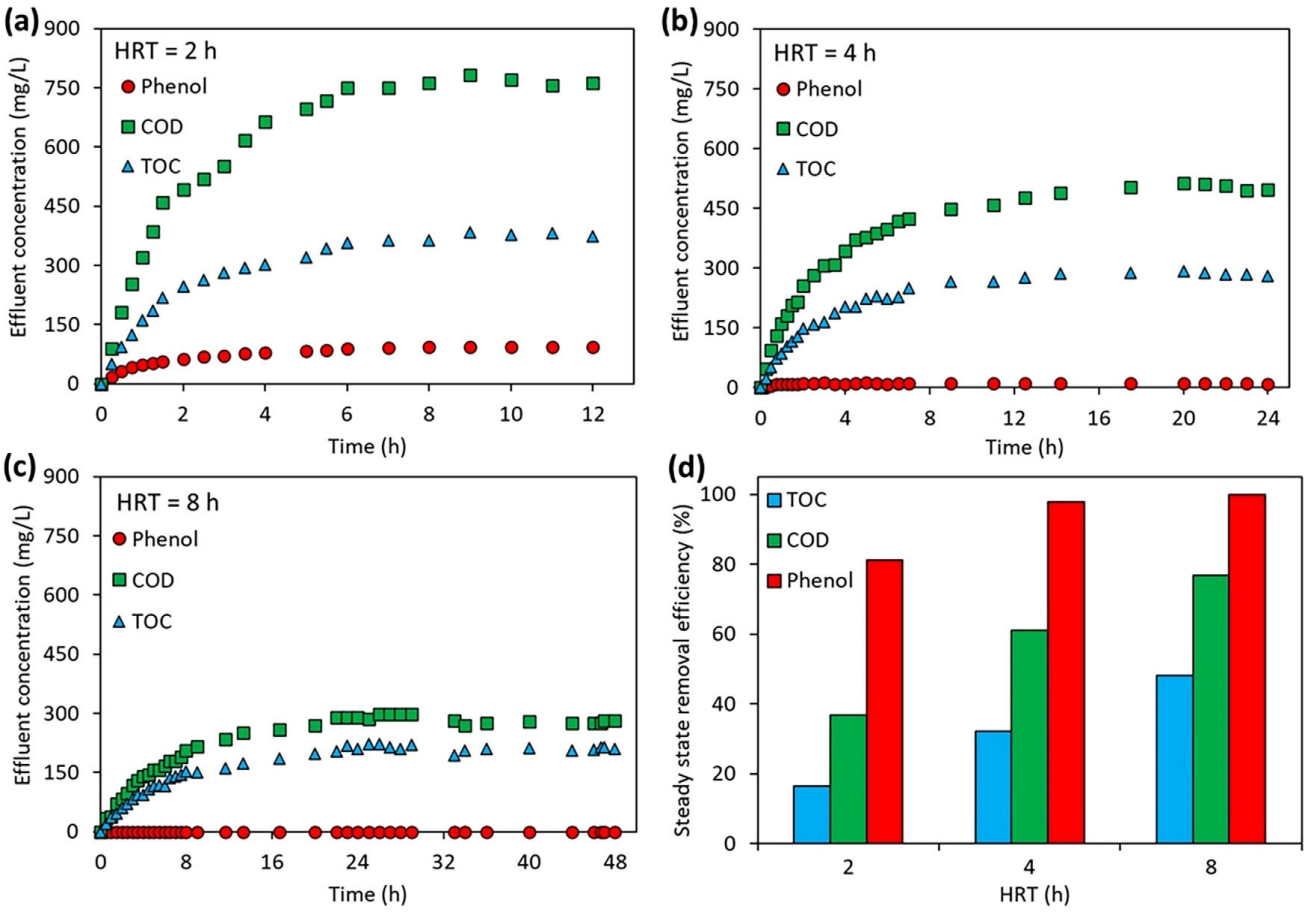
Effect of HRT on the change of a) 2 h, b) 4 h, c) 8 h, and d) steady‐state phenol, COD and TOC removal efficiencies in JLR effluent (influent phenol = 500 mg L^−1^, ozone gas concentration = 17.5 gO_3_ m^−3^).

HRT is an important parameter that directly affects the design, cost, and energy requirements in wastewater treatment.^[^ ^43^ ^]^ In general, higher HRT means higher investment costs. Therefore, the effect of different HRTs on phenol, COD, and TOC removal was investigated. To determine the effect of HRT, ozone gas concentration was kept constant at 17.5 gO_3_ m^−3^ and influent phenol concentration was kept constant at 500 mg L^−1^. Phenol containing solution was fed to the JLR with HRT of 2, 4, and 8 h, respectively. The experimental system was operated in continuous phase until the phenol, COD, and TOC levels in the effluent of the JLR reached steady‐state. Phenol, COD, and TOC changes in the JLR effluent as a result of the experiments are shown in **Figure** **2**. It can be said that ≈7 h is required for phenol, COD, and TOC to reach steady‐state when HRT is 2 h (Figure 2a). When HRT is 4 h, it can be said that phenol values reach steady‐state after about 2 h and COD and TOC values reach steady‐state after about 12 h (Figure 2b). When HRT is 8 h, no phenol is observed in the JLR effluent, while COD and TOC values reach steady‐state after about 22 h (Figure 2c). As can be seen from the results, the time required to reach steady‐state COD and TOC concentrations in the JLR effluent increased with increasing HRT. In general, a shorter HRT resulted in an insufficient reaction time.^[^ ^44^ ^]^ As the HRT is reduced, the organic load entering the JLR increases. The time required for the system to reach steady‐state decreases with the increase in the influent organic load. Therefore, the HRT needs to be optimized in order to obtain high removal efficiencies in a reasonable time. The variation of removal efficiencies obtained for steady‐state condition at different HRTs is shown in Figure 2d. The removal efficiencies of phenol, COD, and TOC at steady‐state for HRT 2 h are 81.1%, 36.8%, and 16.5%, respectively. In the steady‐state condition for HRT 4 h, phenol, COD, and TOC removals are 97.8%, 61.1%, and 32.2%, respectively. For HRT 8 h, phenol is completely removed, while COD and TOC removals are 76.8%, 76.8%, and 48.2%, respectively. When phenol, COD and TOC concentrations in the effluent are evaluated, it is seen that increasing HRT improves the removal efficiencies. This is an expected result as increasing HRT means a longer reaction time. The amount of ozone gas entering the reactor per unit phenol, COD, or TOC under steady‐state conditions at different HRTs was calculated. Longer HRT causes more ozone consumption per unit phenol, COD, or TOC. This results in an increase in removal efficiencies. At 2, 4, and 8 h HRT, 1.25, 2.08, and 3.94 g ozone is required for 1 g phenol removal, respectively. At the same HRT times, 1.11, 1.30, and 2.03 g ozone is consumed for 1 g COD removal, respectively. Similarly, 6.8, 7.4, and 10.2 g ozone are required for 1 g TOC removal, respectively.

### 2.3 Effect of Influent Phenol Concentration

**Figure 3:**
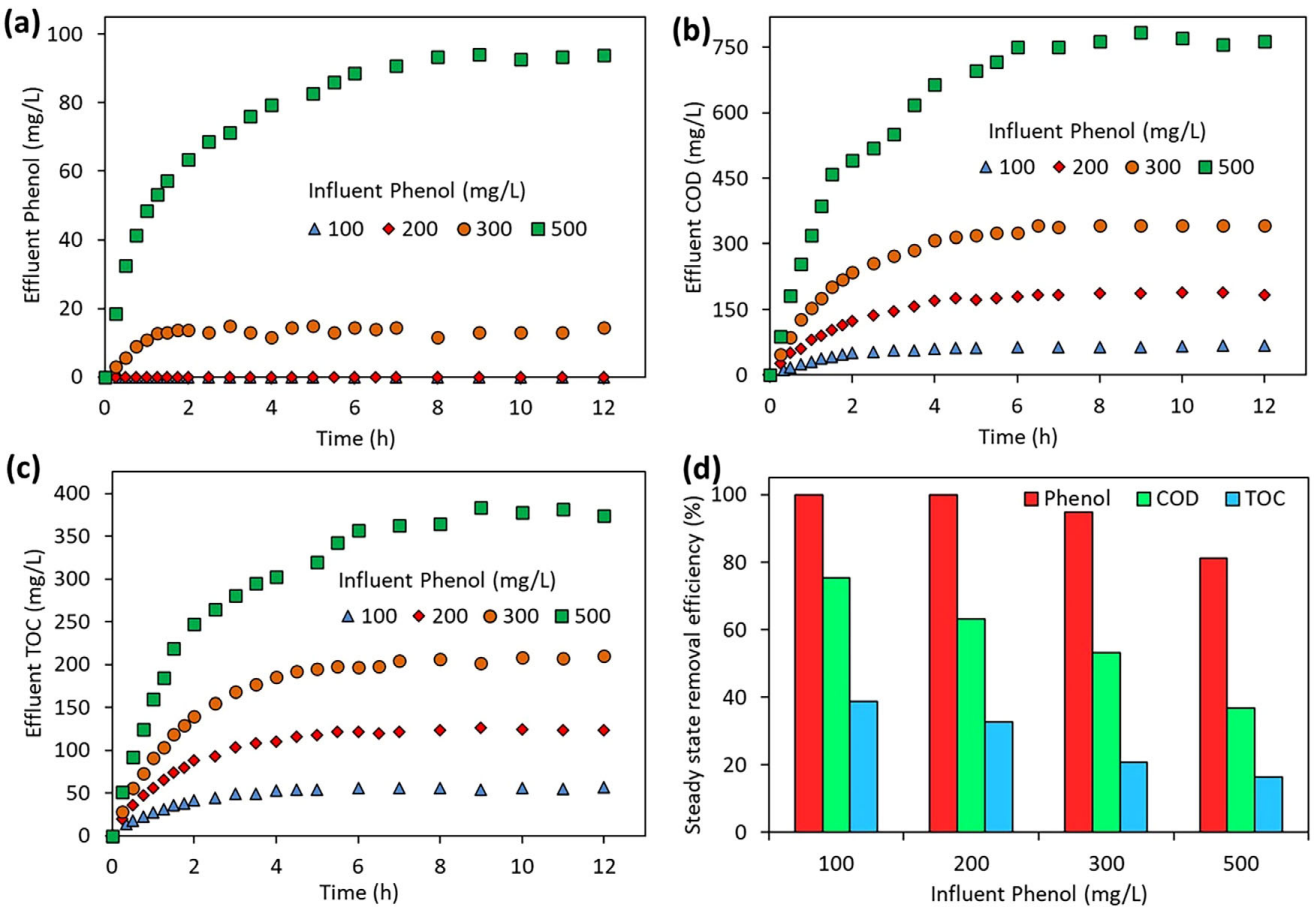
Effect of influent phenol concentration on the change of a) phenol, b) COD, c) TOC, and d) steady‐state phenol, COD, and TOC removal efficiencies in JLR effluent (HRT = 2 h, ozone gas concentration = 17.5 gO_3_ m^−3^).

In order to determine the effect of the influent phenol concentration, synthetic wastewater containing 100, 200, 300, and 500 mg L^−1^ phenol was fed to the JLR operated in continuous phase and the variation of the effluent phenol, COD, and TOC concentrations as a function of the ozonation time was investigated. In the experiments, the HRT was 2 h and the ozone gas concentration was 17.5 gO_3_ m^−3^. **Figure** **3** shows the JLR effluent phenol, COD, and TOC concentrations as a function of influent phenol concentration. At influent phenol concentrations of 100 and 200 mg L^−1^, all phenol was removed, whereas at influent phenol concentrations of 300 and 500 mg L^−1^, the steady‐state effluent phenol removal was 94.9% and 81.1%, respectively (Figure 3a). There is a linear correlation between phenol concentration measured at the influent and COD concentrations (2.495 mg COD/mg phenol), for influent phenol concentrations of 100 and 200 mg L^−1^, COD and TOC were observed in the effluent, although phenol was completely removed. The steady‐state effluent COD removals for 100, 200, 300, and 500 mg L^−1^ influent phenol concentration were measured as 75.3%, 63.2%, 53.1%, and 36.8%, respectively (Figure 3b). A linear correlation exists between phenol concentration and TOC concentrations measured in the JLR influent (0.911 mg TOC∖/mg phenol). Similar to the JLR effluent COD concentration, TOC concentrations also increase in the effluent due to the increase in influent phenol concentrations at steady‐state. With increasing influent phenol concentration, effluent TOC removals were measured as 38.9%, 32.8%, 20.7%, and 16.5%, respectively (Figure 3c). It is observed that the increase in the influent phenol concentration in JLR causes a decrease in the removal efficiencies of phenol, COD, and TOC in the effluent (Figure 3d). COD and TOC removals are at lower levels due to the intermediate products (mainly oxalic acid) that are resistant to ozonation and are formed as a result of the oxidation of phenol by ozonation.^[^ ^41^ ^]^ Although the intermediate products formed are not specified, they are stated to be easily biodegradable in the literature.^[^ ^45^ ^]^ At JLR, the ozonation process can be used as a pretreatment step to reduce the toxic effects of phenol before biological treatment rather than for the complete mineralization of phenol. At different influent phenol concentrations, the amount of ozone entering the reactor per unit phenol, COD, or TOC at steady‐state was calculated. For the removal of 1 g of phenol, ≈ 1.8 g of ozone is required at 300 mg L^−1^ influent concentration, but this amount decreases to 1.3 g at 500 mg L^−1^. The amount of ozone required for the removal of 1 g of COD is 2.37, 1.55, 1.26, and 1.11 g, respectively, with increasing influent COD concentration. Similarly, the amount of ozone required for the removal of 1 g of TOC is determined to be 13.4, 8.1, 7.3, and 6.8 g, respectively, with increasing influent TOC concentration.

## 3 Conclusion

In this study, phenol removal by ozonation in JLR operated in continuous phase under strong alkaline conditions was investigated and the effects of ozone gas concentration, HRT, and influent phenol concentration were evaluated. In steady‐state conditions, removal efficiencies were determined to be in the order of phenol > COD > TOC. It was observed that both COD and TOC removal efficiencies increased with increasing HRT. When HRT was 2 and 4 h, it was determined that 7 and 2 h were required for the effluent phenol concentration to become steady‐state, respectively. When HRT was 8 h, phenol was not detected in the JLR effluent. It was determined that increasing influent phenol concentration decreased the removal efficiencies of phenol, COD and TOC in the effluent. These results demonstrate that ozonation in the JLR is highly effective for phenol removal, achieving complete elimination of phenol from the effluent under optimized conditions. However, the persistence of intermediate oxidation products, particularly oxalic acid, leads to residual COD and TOC values, suggesting that complete mineralization cannot be achieved under the current operating conditions. Therefore, rather than being used as the sole treatment method, ozonation in the JLR can be considered a promising pretreatment step to reduce the toxicity of phenolic compounds to facilitate biodegradability in later treatment stages. Future research should investigate the integration of JLR‐based ozonation with biological treatment systems under different loading conditions in order to develop more sustainable strategies for the treatment of phenol‐containing wastewater. In addition, the effects of solution pH on phenol oxidation and the types of intermediates formed should be investigated in detail. Comprehensive cost and energy analyses are also required to assess the suitability of the JLR system for large‐scale applications.

## 4 Experimental Section

### Chemicals

Phenol, used to prepare phenol‐containing aqueous solutions at JLR and NaOH, used for pH adjustment, were purchased from Merck. Deionized water was used to prepare synthetic wastewater containing phenol. All chemicals and reagents used for COD, TOC, and phenol analyses in effluent samples were of analytical grade or higher.

### Experimental Setup

**Figure 4:**
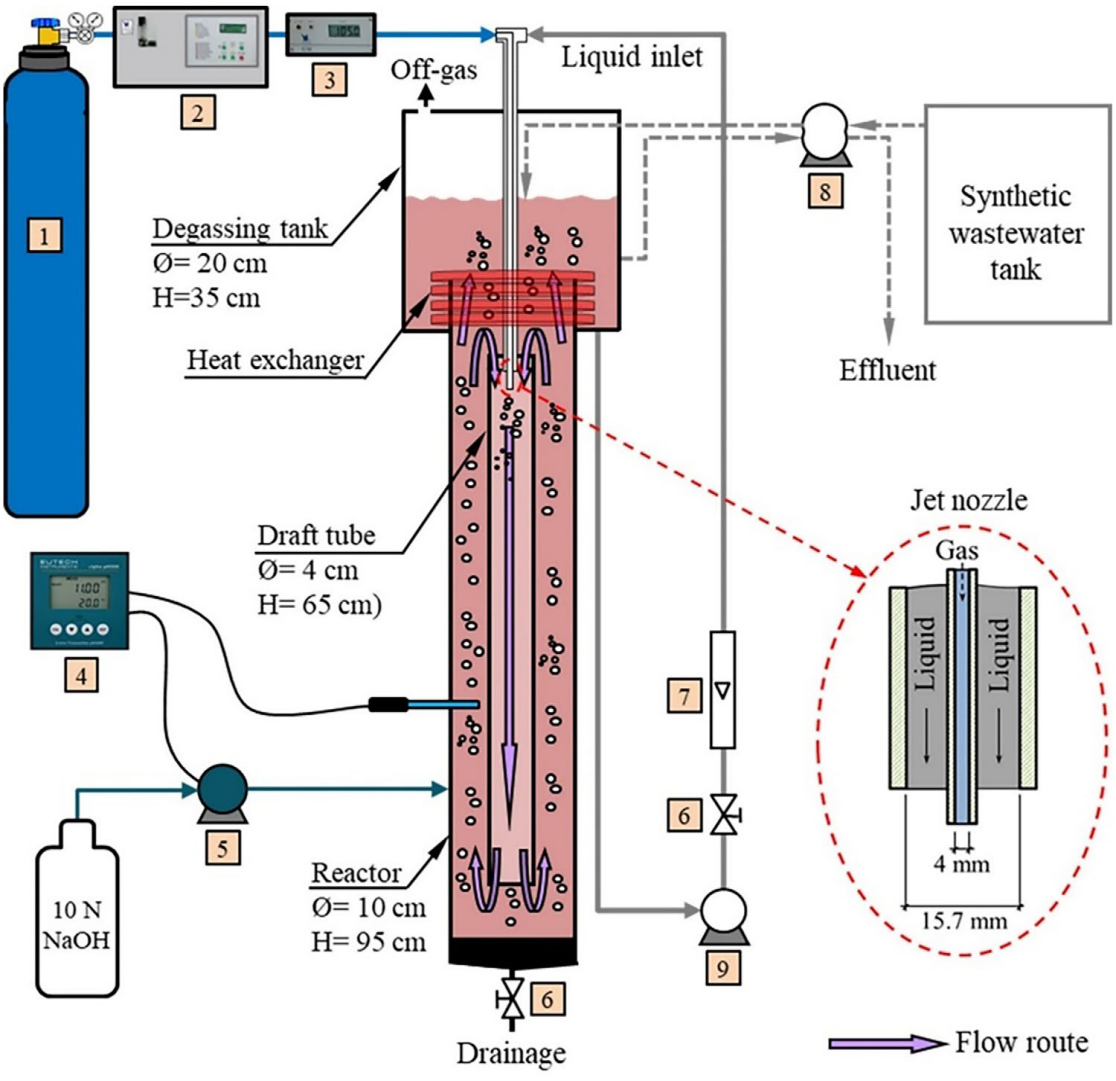
Experimental system schematic diagram (1) Dry air/oxygen bottle, (2) Ozone generator, (3) Ozone analyzer, (4) pH controller, (5) Peristaltic pump (1 head), (6) Valve, (7) Flowmeter, (8) Peristaltic pump (2 heads), (9) Centrifugal pump.

The stainless steel JLR consists of two concentric cylindrical pipes. The reactor (outer) and suction pipe (inner) have internal diameters of 10 cm and 4 cm, respectively. The larger compartment at the top of the reactor is called the degassing tank (height 35 cm, internal diameter 20 cm). Total height including degassing tank is 110 cm. The suction pipe (height 65 cm) is positioned 10 cm above the bottom of the reactor (impact plate) and centered on the reactor by means of a support. Liquid pumped by a circulation pump and gas from a separate line are sprayed into the suction pipe through a jet nozzle. The two‐phase stream traveling in the suction pipe moves downwards and hits the impact plate at the bottom of the reactor and rises between the wall of the reactor and the suction pipe. Part of the liquid, which reaches the suction pipe level and contains air bubbles, is drawn back into the suction pipe due to the pressure difference and the cycle continues. The jet nozzle consists of two concentric pipes. In the jet nozzle, the outer pipe through which the liquid flows has an inner diameter of 15.7 mm and the inner pipe through which the gas passes has an inner diameter of 4 mm and a wall thickness of 1.2 mm. In the JLR, the liquid circulation rate was maintained at 1 L s^−1^, and 18 L synthetic phenol‐containing wastewater was used, prepared with deionized water. In the experimental setup, the hydraulic retention time (HRT) was adjusted by varying the influent flow rate while keeping the reactor volume constant. The temperature was kept constant at 20°C using a serpentine placed in the degassing chamber through which tap water was passed. The pH value in the reactor was controlled at pH 11 using 10 M NaOH. The ozonation experiments have been carried out in the experimental system as shown in **Figure** **4**.

### Analysis

Phenol and COD measurements were analyzed using a UV‐vis spectrophotometer (Spectroflex 6600, WTW) according to the colorimetric method specified in the Standard Methods.^[^ ^46^ ^]^ Phenol analysis was determined by 4‐aminoantipyrine in the presence of ferricyanide at 500 nm (Standard Methods: 5530 D). COD analysis was determined at 600 nm using closed reflux method (Standard Methods: 5220 D). TOC analyses were determined by high temperature combustion method in Tekmar‐Dorhman Apollo 9000 (Standard Methods: 5310 B). The steady‐state condition in the JLR system was determined according to the criterion that the JLR effluent pollutant concentration remains constant within a 5% change range. This tolerance range was taken as the basis for the evaluation of the transition to steady‐state, considering the analytical measurement uncertainties and natural fluctuations in the system.

The ozone gas supplied to the JLR was produced from dry air using an ozone gas generator (COM‐AD‐08, Anseros). Ozone gas was supplied to the JLR with a gas flow rate of 250 L h^−1^. Different ozone dosages were adjusted by varying the operating capacity of the ozone generator at constant gas flow rates. Ozone concentration in the gas phase was measured using non‐dispersive ultraviolet (NDUV) absorption technology (GM‐6000‐OEM, Anseros). Solution pH values were measured and controlled using a pH controller (Alpha pH500, Eutech Instruments).

## Conflict of Interest

The authors declare no conflict of interest.

## Author Contributions

M.S.B. contributed to the investigation, methodology, writing – original draft, and writing – review and editing. I.C. contributed to conceptualization, investigation, methodology, and writing – review and editing. N.D. contributed to conceptualization, investigation, methodology, visualization, writing – original draft, and writing – review and editing. E.Y. led the conceptualization and was responsible for funding acquisition, project administration, investigation, methodology, supervision, resources, visualization, writing – original draft, validation, and writing – review and editing. All authors contributed to revising the manuscript and agree with its published version.

## Data Availability

The data that supports the findings of this study are available from the corresponding author, upon reasonable request.
